# Association of single nucleotide polymorphisms with dyslipidemia and risk of metabolic disorders in the State of Qatar

**DOI:** 10.1002/mgg3.2178

**Published:** 2023-05-05

**Authors:** Dalal Al‐Sharshani, Dinesh Velayutham, Muthanna Samara, Reham Gazal, Ayman Al Haj Zen, Mohamed A. Ismail, Mahmoud Ahmed, Gheyath Nasrallah, Salma Younes, Nasser Rizk, Sara Hammuda, M. Walid Qoronfleh, Thomas Farrell, Hatem Zayed, Palli Valapila Abdulrouf, Manar AlDweik, John Paul Ben Silang, Alaa Rahhal, Rana Al‐Jurf, Ahmed Mahfouz, Amar Salam, Hilal Al Rifai, Nader I. Al‐Dewik

**Affiliations:** ^1^ Heart Hospital (HH) Hamad Medical Corporation (HMC) Doha Qatar; ^2^ Genomics and Precision Medicine (GPM), College of Health & Life Science (CHLS) Hamad Bin Khalifa University (HBKU) Doha Qatar; ^3^ Liberal Arts and Science (LAS) Hamad Bin Khalifa University (HBKU) Doha Qatar; ^4^ Department of Psychology Kingston University London Kingston upon Thames London UK; ^5^ Department of Research, Women's Wellness and Research Center (WWRC) Hamad Medical Corporation (HMC) Doha Qatar; ^6^ College of Health & Life Science (CHLS) Hamad Bin Khalifa University (HBKU) Doha Qatar; ^7^ Hamad Medical Corporation (HMC) Doha Qatar; ^8^ Department of Mathematics, Statistics and Physics, College of Arts and Sciences Qatar University (QU) Doha Qatar; ^9^ Department of Biomedical Science, College of Health Sciences, Member of QU Health Qatar University (QU) Doha Qatar; ^10^ Research & Policy Division Q3CG Research Institute (QRI) 7227 Rachel Drive Ypsilanti Michigan USA; ^11^ 21HealthStreet Company London UK; ^12^ Department of Cardiology, Al Khor Hospital (AKH) Hamad Medical Corporation (HMC) Doha Qatar; ^13^ Neonatal Intensive Care Unit (NICU), Newborn Screening Unit, Department of Pediatrics and Neonatology, Women's Wellness and Research Center (WWRC) Hamad Medical Corporation (HMC) Doha Qatar; ^14^ Faculty of Health and Social Care Sciences, Kingston University St. George's University of London London UK; ^15^ Translational and Precision Medicine Research, Women's Wellness and Research Center (WWRC) Hamad Medical Corporation (HMC) Doha Qatar

**Keywords:** cardiovascular disease (CVD), coronary artery disease (CAD), diabetes, dyslipidemia, hypertension, metabolic, non‐alcoholic fatty liver disease (NAFLD), Qatar genome project (QGP), single nucleotide polymorphism (SNP)

## Abstract

**Background:**

Dyslipidemia is recognized as one of the risk factors of cardiovascular diseases (CVDs), type 2 diabetes mellitus (T2DM), and non‐alcoholic fatty liver disease (NAFLD).

**Objective:**

The study aimed to investigate the association between selected single nucleotide polymorphisms (SNPs) with dyslipidemia and increased susceptibility risks of CVD, NAFLD, and/or T2DM in dyslipidemia patients in comparison with healthy control individuals from the Qatar genome project.

**Methods:**

A community‐based cross‐sectional study was conducted among 2933 adults (859 dyslipidemia patients and 2074 healthy control individuals) from April to December 2021 to investigate the association between 331 selected SNPs with dyslipidemia and increased susceptibility risks of CVD, NAFLD and/or T2DM, and covariates.

**Results:**

The genotypic frequencies of six SNPs were found to be significantly different in dyslipidemia patients subjects compared to the control group among males and females. In males, three SNPs were found to be significant, the rs11172113 in over‐dominant model, the rs646776 in recessive and over‐dominant models, and the rs1111875 in dominant model. On the other hand, two SNPs were found to be significant in females, including rs2954029 in recessive model, and rs1801251 in dominant and recessive models. The rs17514846 SNP was found for dominant and over‐dominant models among males and only the dominant model for females. We found that the six SNPs linked to gender type had an influence in relation to disease susceptibility. When controlling for the four covariates (gender, obesity, hypertension, and diabetes), the difference between dyslipidemia and the control group remained significant for the six variants. Finally, males were three times more likely to have dyslipidemia in comparison with females, hypertension was two times more likely to be present in the dyslipidemia group, and diabetes was six times more likely to be in the dyslipidemia group.

**Conclusion:**

The current investigation provides evidence of association for a common SNP to coronary heart disease and suggests a sex‐dependent effect and encourage potential therapeutic applications.

## Graphical abstract

We investigated the association of single nucleotide polymorphisms with dyslipidemia, cardiovascular diseases, type 2 diabetes mellitus, and non‐alcoholic fatty liver disease.

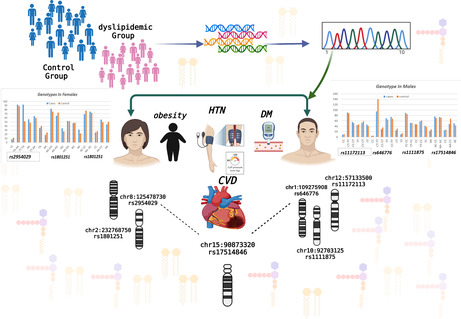

## 1 INTRODUCTION

Dyslipidemia is a polygenic condition caused by a combination of both genetic and environmental factors. There is a growing body of evidence suggesting that dyslipidemia is a contributing factor in the pathogenesis of cardiovascular diseases (CVDs), type 2 diabetes mellitus (T2DM), and non‐alcoholic fatty liver disease (NAFLD) (Cui et al., 2020; Lai et al., 2005; Liu et al., 2019; Sandhu et al., 2008; Wu et al., 2016). Multiple genetic loci associated with blood lipid levels have been discovered by genome‐wide association studies (GWAS), with 157 loci significantly associated with lipid levels. These included 62 novel loci from which 24 loci were highly related to high‐density lipoprotein‐cholesterol (HDL‐C), 15 loci with low‐density lipoprotein‐cholesterol‐C (LDL‐C), 8 loci with triglyceride (TG) levels, and 15 loci with total cholesterol (TC). In addition, GWAS has identified a number of candidate loci conferring risk or protection from CVD including coronary artery disease (CAD) and myocardial infarction (MI). For instance, dyslipidemia and MI were found to have a significant association with several polymorphisms such as rs599839 on human chromosome 1p13.3, rs974819 in the *PDGFD* gene, several polymorphisms in *TNF‐α*, and rs4977574 of the *CDKN2BAS* gene (Hou et al., 2009; Huang et al., 2014; Kleber et al., 2010; Rizk et al., 2015; Zhou et al., 2012). Several studies reported that young people with CVD had a higher genetic burden of single nucleotide polymorphisms (SNPs) than their older counterpart; and some SNPs had pleiotropic effects on the following factors including CAD, body mass index (BMI), C‐reactive protein, blood pressure, lipids, and T2DM (Andersson et al., 2019; Khera et al., 2016; Tada et al., 2016; Waziri et al., 2016). The prevalence of metabolic syndrome components in Qatar is significantly greater than in other countries. The International Diabetes Federation (IDF) published data in 2021 on the prevalence of T2DM and estimated that 537 million adults between the ages of 20–79 years are living with diabetes worldwide, while in the Middle East and North Africa (MENA region) 1 in 6 adults (ca. 73 million) are living with diabetes (IDF; 2021). In Qatar alone, the age‐adjusted comparative prevalence of diabetes currently stands at 19.5% and it is predicted to rise to almost 23% by 2045 (Magliano, 2021). Data concerning obesity prevalence in Qatar is scant. The Qatar National STEPwise Survey of the population found that 41% of the participants aged 18–64 years were categorized as obese (BMI ≥30 kg/m^2^), with women (43%) having a slightly higher obesity prevalence than men (40%) (Haj Bakri & Al‐Thani, 2013). A recent analysis that used electronic medical records from primary care (*n* = 176,170; age ≥18 years) reported obesity prevalence as 33%, however, the study did not differentiate between indigenous Qatari and non‐Qatari populations (Taheri & Al‐Thani, 2021). As a result, current trends show large increases in the population segment that potentially fit the metabolic syndrome criteria, which would increase population vulnerability to more severe health issues as well as premature mortality (Syed et al., 2020). Metabolic syndrome is a complex pathophysiological condition characterized by the co‐occurrence of insulin resistance, hypertension, atherogenic dyslipidemia, and obesity (Jallow et al., 2019).

In general, having more than three of these etiologically linked cardiometabolic hazards can raise the likelihood of developing several chronic illnesses such as cancer, schizophrenia, chronic kidney disease, arthritis, T2DM and CVD, and premature mortality (Lee et al., 2017). Nirwan and Singh (2021) showed that males had higher mean lipid levels and a higher prevalence of atherogenic dyslipidemia than women of a younger age. A study in 64 hospitals from the gulf region (Bahrain, Oman, Qatar, Kuwait, the UAE, and Yemen) revealed that dyslipidemia prevalence was higher in females (44%) than males (28%) (Aljefree & Ahmed 2015; El‐Menyar et al., 2009, 2011). Likewise, the Saudi Project for Assessment of Coronary Events (SPACE) reported that the prevalence of dyslipidemia is 31% among acute coronary syndrome patients (Aljefree & Ahmed 2015). Despite the wide variation across research that might be attributable to the population type analyzed, the incidence of dyslipidemia in the Middle East appears to be considerable. This feature, however, mandates the employment of a common definition for an appropriate assessment of the size of dyslipidemia in the Middle East region as well as in Qatar (Nirwan & Singh, 2021).

Numerous studies have identified common genetic variations associated with dyslipidemia and increased susceptibility risk of CVD (e.g., rs11172113, rs646776, rs1111875, rs1801251), NAFLD (e.g., rs2954029), T2DM (e.g., rs1111875), and early‐stage coronary atherosclerosis (e.g., rs17514846, rs9349379) in different ethnic groups (Deloukas et al., 2013; Dichgans et al., 2014; Gornik et al., 2019; Hou et al., 2009; Huang et al., 2014; Kiando et al., 2016; Kleber et al., 2010; Liu, et al., 2019; Nikpay et al., 2015; Ren et al., 2017; Rizk et al., 2015; Turley et al., 2020; Webb et al., 2017; Yamasaki et al., 2022; Zhao et al., 2018; Zhou et al., 2012). Although genetic variants associated with dyslipidemia have been extensively studied in European populations, the genes, which confer susceptibility to dyslipidemia in Middle Eastern populations remain to be elucidated. Therefore, in the present study, we aim to investigate the association of genetic variants with dyslipidemia and the risk of metabolic disorders in Qatar while taking into account demographic and biomedical factors.

## 2 MATERIALS AND METHODS

### 2.1 Study design and participants

A community‐based cross‐sectional study was conducted among 2933 adults (Mean age = 38.96 ± 14.16 years) residents in the State of Qatar from April to December 2021. The participants were divided into two groups, the dyslipidemia group (*N*: 859) (Mean age = 37.9 years; SD: 12.2) and healthy control group with no dyslipidemia (*N*: 2074) (Mean age = 42 years; SD: 11.1). The participants did not have inherited causes such as low‐density lipoprotein (LDL) mutation.

The study was approved by the Institutional Review Boards of the Qatar Biobank Protocol no. QF‐QBB‐RES‐ACC‐00018.

### 2.2 Measurements

In this study, dyslipidemia was defined as having any one of the following: serum TC level ≥6.21 mmol/L, TG serum TG level ≥2.31 mmol/L, high LDL‐C (serum LDL‐C level ≥4.10 mmol/L), low HDL‐C (serum HDL‐C level <1.0 mmol/L), use of antihyperlipidemic medications, or self‐reported dyslipidemia.

The study also included demographic factors (gender and age) and medical factors. The medical factors included firstly, obesity, which was defined as BMI ≥30 kg/m^2^ while central obesity was defined according to the WHO criteria (WHO, 2011). Secondly, lipid profiles where waist‐to‐hip ratio of ≥0.90 for men and ≥0.85 for women were considered. Thirdly, hypertension was defined as systolic blood pressure ≥140 mmHg with or without diastolic blood pressure ≥90 mmHg, or a previous diagnosis of hypertension. Fourth, diabetes was defined as random blood glucose ≥11.1 mmol/L or glycated hemoglobin (HbA1c) ≥6.5% as described elsewhere (Liu et al., 2019; Qiu et al., 2020).

### 2.3 Single nucleotide polymorphisms selection

A total of 331 well‐known SNPs, which were found to be significantly associated with dyslipidemia, CAD, obesity, and T2DM in previous studies were investigated (Abdullah et al., 2021; Alathari et al., 2020; Broadbent et al., 2008; Bryant et al., 2013; Cooke et al., 2012; Gkouskou et al., 2022; Lazzaretti et al., 2013; Li et al., 2020; McClung et al., 2019; Takeuchi et al., 2011; Tekola‐Ayele et al., 2013; Webb et al., 2017; Zhou et al., 2012) (Table S1) using GWAS that have become increasingly popular to identify associations between SNPs and phenotypic traits (Marees et al., 2018). The genetic screening for the 331 SNPs was done on the entire genome.

### 2.4 Association analysis, covariates and interactions, and statistical analysis

We have performed routine quality check for sample quality, call rate, sex bias, and Hardy Weinberg (Marees et al., 2018). The total samples were used for analysis is 2933 and the total genotype rate was 0.999523. For comparison of the allele and genotype frequencies of these SNPs among dyslipidemia patients and control, Chi‐square, odds ratios (ORs), and 95% confidence interval (CI) were calculated and reported. The statistical significance was set at *p* ≤ 0.05. Logistic regression models (co‐dominant, dominant, and recessive) were conducted before and after adjusting for covariates (gender, obesity, hypertension, and diabetes). The association analysis used a full logistic model comprising three genetic effects: additive effects of allele dosage (ADD), dominance deviation from additivity (DOMDEV) with negative indicating that the allele is recessive, and 14‐df joint test of both additive and dominance (GENO_14DF) that adds genotype x covariate interactions to the model. The QC, statistical analyses, and genetic modeling were performed with SAS and PLINK 2.0.

## 3 RESULTS

### 3.1 Demographics and clinical characteristics

Out of the 2933 subjects included in the present study, 29.3% (859/2933) were found to have dyslipidemia (female to male ratio: 1:2; age 42 ± 11.1 years), whereas 70.7% (2074/2933) did not have dyslipidemia and thus, were included in the control group (female to male ratio: 1:1; age 37.9 ± 12.2 years). Patients' demographics and clinical characteristics along with the health status and comorbidities of obesity, hypertension, and diabetes among dyslipidemia and control groups are shown in Table 1.

**TABLE 1:** Basic characteristics of the study population of dyslipidemia and control participants (*n* = 2933).

Characteristics	Dyslipidemia	Control	OR (95% CI)	*p* value
	*n* (%)	*n* (%)		
	(*n* = 859)	(*n* = 2074)		
Gender				
Female	260 (30.26%)	1202 (57.95%)	Reference	
Male	599 (69.73%)	872 (42.04%)	3.17 (2.68–3.76)	<0.0001
Obesity^a^				
Yes	369/855 (43.16%)	739/2070 (35.70%)	1.36 (1.16–1.60)	0.00015
No	486/855 (56.84%)	1331/2070 (64.30%)	Reference	
Hypertension^a^				
Yes	117/734 (15.94%)	168/1890 (8.9%)	1.94 (1.50–2.50)	<0.0001
No	617/734 (84.06%)	1722/1890 (91.11%)	Reference	
Diabetes^a^ status				
Yes	151/327 (46.17%)	176/1582 (11.13%)	6.85 (5.24–8.96)	<0.0001
No	176/327 (53.82%)	1406/1582 (88.87%)	Reference	

^a^ Denotes missing data and therefore the denominator was reported accordingly.

Individuals in the dyslipidemia group were significantly more likely to be males, obese, hypertensive, and diabetic compared with the control group (Table 1).

### 3.2 Genetic findings

Out of 331 SNPs studied, six SNPs were found to be significantly different in dyslipidemia patients in comparison with the control group. These were rs11172113, rs646776, rs1111875 in males, and rs1801251 and rs2954029 in females, and rs17514846 in both groups (Tables 2 and 3).

**TABLE 2:** Six significant SNPs among dyslipidemia patients.

Genomic coordinate	Gene	Rs no.	Amino acid	Dyslipidemia groups
12:57133500	*LRP1*	rs11172113	—	Males
1:109275908	*PSRC1/ CELSR2*	rs646776	—	Males
10:92703125	*HHEX*	rs1111875	—	Males
2:232768750	*KCNJ13 & GIGYF2*	rs1801251	p. Thr175Ile	Females
8:125478730	*TRIB1*	rs2954029	—	Females
15:90873320	*FURIN*	rs17514846	—	Both

**TABLE 3:** Comparison of genotypic and allelic distribution of the six SNPs in dyslipidemia (females/males) compared to controls.

SNP ID (males)	Genotype	Cases	(%)	Control	(%)	*p* value	Allele	Cases	Control	*p* value
		(*N* = 599)		(*N* = 872)						
rs11172113	CC	53	8.85	102	11.7	**0.049**	C	386	562	0.998
	CT	280	46.74	358	41.06		T	812	1182	
	TT	266	44.41	412	47.25					
rs646776	CC	28	4.67	31	5.18	**0.013**	C	206	340	0.115
	CT	150	25.04	278	31.88		T	992	1404	
	TT	421	70.28	563	64.56					
rs1111875	TT	62	10.35	130	14.91	**0.027**	T	376	591	0.155
	TC	252	42.07	331	37.96		C	822	1153	
	CC	285	47.58	411	47.13					
rs17514846	AA	137	22.87	253	29.01	**0.005**	A	581	883	0.255
	AC	307	51.25	377	43.23					
	CC	155	25.88	242	27.75		C	617	861	

SNP ID (females)	Genotype	Cases	(%)	Control	(%)	*p* value	Allele	Cases	Controls	*p* value
		(*n* = 260)		(*n* = 1202)						
rs2954029	TT	20	7.69	136	11.31	**0.038**	T	133	756	**0.008**
	TA	93	35.77	484	40.27					
	AA	147	56.54	582	48.42		A	387	1648	
rs1801251	AA	47	18.08	296	24.63	**0.016**	A	217	1173	**0.003**
	AG	123	47.31	581	48.34					
	GG	90	34.62	325	27.04		G	303	1231	
rs17514846	AA	81	31.15	264	21.96	**0.006**	A	277	1133	**0.011**
	AC	115	44.23	605	50.33		C	243	1271	
	CC	64	24.62	333	27.70					

As shown in (Table 3) the comparison of allelic and genotypic frequencies between dyslipidemia and control groups showed that there were significant differences in the allelic frequencies for three SNPs rs2954029, rs1801251, and rs17514846 and significant differences in the genotypic frequencies for the six SNPs between the two groups.

Among males, three SNPs s11172113, rs646776, and rs1111875 were found to be genotypically significant. Table 3 also shows the frequencies of each variant across the three genotypes including C/C, C/T, and T/T among the dyslipidemia and control groups.

In the female group, two SNPs rs2954029 and rs1801251 were found to be genotypically significant (*p* values = 0.038 and 0.016, respectively). The genotypic frequencies of each variant are shown in Table 3.

In both genders, the rs17514846 was found to be significant. In males, the genotypic frequencies were 22.87% for A/A, 51.25% for A/C and 25.88% for C/C in the dyslipidemia group, and 29.01% for A/A, 43.23% for A/C, and 27.75% for C/C in the control group. In females, the genotypic frequencies were as follows, 31.15% for A/A, 44.23% for A/C, and 24.62% for C/C in the dyslipidemia group, and 21.96% for A/A, 50.33% for A/C, and 27.70% for C/C in the control group.

In comparison with the control group in model studies for males, the rs11172113, rs646776, and rs1111875 were found to be significant. The rs11172113 was found for the over‐dominant (CC‐TT/CT: OR = 1.26, 95% CI = (1.02–1.55), *p* = 0.030); the rs646776 for the over‐dominant (CC‐TT/CT: OR = 0.71, 95% CI = (0.56–0.90), *p* = 0.004) and the recessive (CC‐CT/TT: OR = 1.29, 95% CI = (1.03–1.62), *p* = 0.021) models, and the rs1111875 was found for the dominant model (TT/TC‐CC: OR = 1.51, 95% CI = (1.09–2.94), *p* = 0.010).

On the other hand, the rs2954029 and rs1801251 were found to be significant in females. The rs2954029 for the recessive model (TT‐TA/AA: OR = 1.53, 95% CI = (1.21–1.93), *p* = 0.0003) while the rs1801251 was found to be significant in the dominant (AA/AG‐GG: OR = 1.48, 95% CI = 1.05–2.08, *p* = 0.023), and the recessive (AA‐AG/GG: OR = 1.42, 95% CI = (1.07–1.90), *p* = 0.013) models (Table 4).

**TABLE 4:** Logistic regression of the SNPs in dyslipidemia (females/males) compared to controls.

SNP ID (males)	Model	Genotype	Cases	(%)	Control	(%)	OR (95% CI)	*p* value
			(*n* = 599)		(*n* = 872)			
rs11172113	Dominant	CC	53	8.85	102	11.70	Reference	
		CT‐TT	546	91.15	770	88.30	1.36 (0.96–1.93)	0.080
	Recessive	CC‐CT	333	55.59	460	52.75	Reference	
		TT	266	44.41	412	47.25	0.89 (0.72–1.09)	0.283
	Over‐dominant	CC‐TT	319	53.26	514	58.94	Reference	
		CT	280	46.74	358	41.06	1.26 (1.02–1.55)	**0.030**
rs646776	Dominant	CC	28	4.67	31	3.56	Reference	
		CT‐TT	571	95.33	841	96.44	0.75 (0.44–1. 26)	**0.281**
	Recessive	CC‐CT	178	29.72	309	35.44	Reference	
		TT	421	70.28	563	64.56	1.29 (1.03–1.62)	**0.021**
	Over‐dominant	CC‐TT	449	74.96	594	68.12	Reference	
		CT	150	25.04	278	31.88	0.71 (0.56–0.90)	**0.004**
rs1111875	Dominant	TT	62	10.35	130	14.91	Reference	
		TC‐CC	537	89.65	742	85.09	1.51 (1.09–2.94)	**0.010**
	Recessive	TT‐TC	314	52.42	461	52.87	Reference	
		CC	285	47.58	411	47.13	1.01 (0.82–1.25)	0.862
	Over‐dominant	TT‐CC	347	57.93	541	62.04	Reference	
		TC	252	42.07	331	37.96	1.18 (0.96–1.46)	0.113
rs17514846	Dominant	AA	137	22.87	253	29.01	Reference	
		AC‐CC	462	77.13	619	70.99	1.37 (1.08–1.75)	**0.008**
	Recessive	AA‐ AC	444	74.12	630	72.25	Reference	
		CC	155	25.88	242	27.75	0.90 (0.71–1.15)	0.427
	Over‐dominant	AA‐CC	292	48.75	594	68.12	Reference	
		AC	307	51.25	377	43.23	1.65 (1.34–2.03)	**0.001**

SNP ID (females)	Model	Genotype	Cases	(%)	Control	(%)		*p* value
			*n* = 260		*n* = 1202			
rs2954029	Dominant	TT	20	7.69	136	11.31	Reference	
		TA‐AA	240	92.31	1066	88.69	1.53 (0.93–2.49)	0.086
	Recessive	TT‐TA	240	92.31	620	51.58	Reference	
		AA	147	56.54	582	48.42	1.53 (1.21–1.93)	**0.0003**
	Over‐dominant	TT‐AA	167	64.23	718	59.73	Reference	
		TA	93	35.77	484	40.27	0.82 (0.62–1.09)	0.178
rs1801251	Dominant	AA	47	18.08	296	24.63	Reference	
		AG‐GG	213	81.92	906	75.37	1.48 (1.05–2.08)	**0.023**
	Recessive	AA‐AG	170	65.38	877	72.96	Reference	
		GG	90	34.62	325	27.04	1.42 (1.07–1.90)	**0.013**
	Over‐dominant	AA‐GG	137	52.69	621	51.66	Reference	
		AG	123	47.31	581	48.34	0.95 (0.73–1.25)	0.764
rs17514846	Dominant	AA	81	31.15	264	21.96	Reference	
		AC‐CC	179	68.85	938	78.04	0.62 (0.46–0.83)	**0.001**
	Recessive	AA‐ AC	196	75.38	869	72.30	Reference	
		CC	64	24.62	333	27.70	0.85 (0.62–1.16)	0.310
	Over‐dominant	AA‐CC	145	55.77	597	49.67	Reference	
		AC	115	44.23	605	50.33	0.78 (0.59–1.02)	0.072

*Note*: Bold values denote statistical significance at the *p* < 0.05 level.

The shared variant rs17514846 was found to be significant in both dyslipidemia male and female groups in comparison with the control groups, in males this was for the dominant (AA/AC‐CC: OR = 1.37, 95% CI = 1.08–1.75, *p* = 0.008), and the over‐dominant (AA‐CC/AC: OR = 1.65, 95% CI = 1.34–2.03, *p* = 0.001) models, whereas for females, it was only significant for the dominant model (AA/AC‐CC: OR = 0.62, 95% CI = 0.46–0.83, *p* = 0.001) (Table 4).

### 3.3 Covariates interaction studies

After adjusting for the four covariates, gender, obesity, diabetes, and hypertension, the difference between dyslipidemia and the control group remained significant for the six variants (Table S2).

## 4 DISCUSSION

Dyslipidemia prevalence varies among countries depending on the ethnic population studied, geographical location, socioeconomic status, gender, and the occurrence of metabolic disorders and genetic factors (Zhang et al., 2012). The proportion of dyslipidemic subjects in our study is 29.3%. In a National Health and Nutrition Examination Survey (NHANES), which was carried out between 1999 and 2002 on 4275 subjects in the USA, the estimated prevalence of dyslipidemia was found to be 52.9%. (Al Rasadi et al., 2016; Bays et al., 2008). In the Multi‐Ethnic Study of Atherosclerosis (MESA, 2000–2002) of six USA communities using the updated NCEP ATP III criteria, the overall dyslipidaemia prevalence was 29.3% (Goff Jr. et al., 2006). In the Canadian Health Measures Survey (2007–2009), the overall dyslipidaemia prevalence was 45% (Joffres et al., 2013). While in the German Metabolic and Cardiovascular Risk Project (GEMCAS) (Steinhagen‐Thiessen et al., 2008), the overall dyslipidemia rate was 76%. The findings from this study (29.3%) falls within the gulf region prevalence range (Aljefree & Ahmed 2015; El‐Menyar et al., 2009, 2011).

Our findings revealed that genetic variations in dyslipidemia were significantly different from those in the healthy population. These differences were found for the rs11172113, rs646776, and rs1111875 variants in males, and the rs1801251 and rs2954029 variants in females, and the rs17514846 variant in both groups.

The rs11172113 variant on chromosome 12q13 locus, is in intron 1 of *LRP1* (LDL receptor‐related protein‐1). This12q13 locus was found to be associated with abdominal aortic aneurysm (Bown et al., 2011). The rs11172113 is a LDL receptor family member that regulates extracellular proteolytic activities and plays a crucial role in mediating inflammation and efferocytosis (Webb et al., 2017). There was an association between the risk allele of CAD at rs11172113 and the reduced expression of LRP1 in atherosclerotic and nonatherosclerotic arterial wall, as well as the expression of quantitative trait loci (eQTLs) in subcutaneous adipose tissue (Webb et al., 2017). In our study, the CT genotype distribution of rs11172113 in *LRP1* was found to be significantly higher in dyslipidemia male cases vs controls. This SNP is known to be associated to CVD and has recently been also found to be associated with fibromuscular dysplasia (FMD) in females (Georges et al., 2021; Turley et al., 2020).

The rs646776 is located on the chromosomal region at 1p13.3 in two genes, the cadherin EGF LAG seven‐pass G‐type receptor 2 (CELSR2) and the proline/serine‐rich coiledcoil 1 (PSRC1). Moreover, it was found to be associated with mRNA expression levels of CELSR2, PSRC1, and sortilin 1 (SORT1 mRNA) (Kathiresan et al., 2008; Musunuru et al., 2010). The association with LDL and cholesterol levels in this region was recently explained by the identification of rs12740374, which is a proxy of rs646776. This SNP alters the expression of SORT1 in the liver and thereby affects the secretion of LDL by liver cells (Musunuru et al., 2010). This association was also found to increase the risk for MI (Deloukas et al., 2013; Rizk et al., 2015). In our study, the TT genotype was found to be significantly higher in dyslipidemia (OR: 1.29; 95% CI: 1.03–1.62), while the CT genotype was found to be significantly higher in control males (OR: 0.71; 95% CI: 0.56–0.90).

The rs1111875 is in chromosome locus 10q23.33 in *HHEX* gene, next to the insulin‐degrading enzyme gene (Morgutti et al., 2001). This gene was found to be involved in glucose and lipid metabolism and in regulating insulin secretion, where it plays important roles in carbohydrate intolerance and diabetes (Tarnowski et al., 2017). It was also found that changes in HHEX expression leading to diabetes (Klimentidis et al., 2014).

The rs1111875 was found to be related to the risk of type 2 diabetes (T2DM) in European and Chinese populations (Hu & Jia, 2018; Li et al., 2020; Wang et al., 2017). It was also found to be associated with early‐onset T2DM (Cai et al., 2011; Li et al., 2020; Pivovarova et al., 2009) and could be a risk factor for CVD. The C allele of rs1111875 of *HHEX* increases susceptibility to T2DM, but this may vary across different ethnic populations (Cai et al., 2011; Pivovarova et al., 2009; van Vliet‐Ostaptchouk et al., 2008). In another study, the *HHEX* rs1111875 haplotype analysis showed that rs1111875 C was associated with the development of T2DM (Li et al., 2020). Our study showed that the TC‐CC genotype is significantly higher in males with dyslipidemia compared with controls and may increase the risk of T2DM in dyslipidemia cases.

The rs17514846 SNP chromosomal location is 15q26.1, which includes the oncogene FES upstream region (FURIN). FURIN is a serine proteinase that is widely expressed in tissues of many organs including the heart and is largely concentrated in the trans‐Golgi network (Thomas, 2002). Functionally, FURIN mediates protein processing as it cleaves and activates many precursor proteins. For instance, FURIN is essential for heart development as its absence causes cardiac malformation and early postnatal death (Kim et al., 2012). In vitro, the minor allele A of this SNP was found to be associated with higher FURIN expression in human umbilical vein endothelial cells (Yang et al., 2020) and mononuclear cells (Zhao et al., 2018). Importantly, it is reported that the rs17514846 SNP is associated with dyslipidemia (Abe et al., 2015) and with metabolic syndrome (Ueyama et al., 2015). Moreover, FURIN promotes arteriosclerosis through the activation of matrix metalloproteinase (MT‐MMP1), TGF‐beta1 (TGF‐β1), and ET‐1 (Stawowy et al., 2004). The rs17514846 has been proposed to be a potential risk for CAD (Zhao et al., 2018).

A recent study showed that *FURIN* SNP rs17514846 was associated with an increased risk of early‐stage coronary atherosclerosis in human cardiac specimens (Ren et al., 2017; Yamasaki et al., 2022; Zhao et al., 2018). FURIN level in the myocardium of cases with the AA was higher than that in CC cases. In our study, the AC‐CC and AC genotypes were found to be significantly higher in the male dyslipidemia group. While for the AC‐CC genotype it was higher in the female control group and AC genotype. This SNP can be a risk for early‐stage coronary atherosclerosis in males and probably a protective SNP in females.

The rs2954029 is an intronic variant in the *TRIB1* gene, which is located on chromosome 8 (8q24). This variant was found to be associated with cardiometabolic phenotypes cholesterolemia, triglyceridemia, CAD, and other metabolic diseases (Deloukas et al., 2013; Jadhav & Bauer, 2019).

*TRIB1* encodes the protein tribbles homolog one that is involved in many human diseases, including myeloid leukemia, Crohn's disease, NAFLD, dyslipidemia, and CAD (Bauer et al., 2015), which is part of mitogen‐activated protein kinases and may regulate lipid metabolism (Hegedus et al., 2007; Vilkeviciute et al., 2019). Furthermore, TRIB1 was found to play an essential role in CAD and atherosclerosis through its overexpression (Sung et al., 2007). It is believed that it controls chemotaxis and proliferation of smooth muscle cells in the arterial intima and lead to atherosclerosis (Varbo et al., 2011; Zhang et al., 2011).

The rs2954029 A allele in a *TRIB1* increases the serum lipid profiles. In addition, rs2954029 A carriers possess higher serum levels of GGT, TG, TC, and LDL (Liu et al., 2019). In our study, the genotype distribution of rs2954029 in *TRIB1* was significantly different between dyslipidemia female patients and healthy controls. This is similar to a previous research in the Chinese Han population but not sex‐dependent (Liu et al., 2019). In the same study, the rs2954029 was found to be significantly associated with the risk of NAFLD in the Chinese Han population, the TA and AA genotypes of rs2954029 were found to significantly increase the risk of NAFLD (Liu et al., 2019). Whereas in our study, we found that only the AA genotype was significant among dyslipidemia and may increase the risk of NAFLD (OR = 1.53; 95% CI: 1.21–1.93). In Malay population this SNP was found to be significantly associated with elevated levels of TC and LDL (Tai et al., 2009) The rs1801251 variant (2q37.1) in GWAS maps to two genes, *KCNJ13* and *GIGYF2*. The functional importance and the relationship of these two genes to CVD is not clear. This variant was significantly associated with CVD (Webb et al., 2017). Our study showed that AG‐GG and GG were significantly associated with dyslipidemia in females and may increase the risk of CVD.

The study investigates the genetic predisposition to dyslipidemia, however, environmental factors (e.g., health‐related lifestyle and behaviours) can also affect the development of dyslipidemia. Future studies should take into account the interaction of genetic‐environmental factors and using precision medicine approach (Al‐Dewik et al., 2022; Al‐Dewik & Qoronfleh, 2019) and population health analyses (Zhai et al., 2023).

## 5 CONCLUSIONS

The results show that the ratio of dyslipidemia was observed more in males than females. The main characteristics of dyslipidemia were obesity, hypertension, and diabetes, which can be linked to CVD. Our findings documented genetic variations that were significantly associated with dyslipidemia, and the risk of developing CVD, NAFLD, and T2DM in Qatari population for the first time. More research in a larger and multi‐ethnic populations is required to validate the current findings.

Our study also provides evidence of association for common SNPs to CVDs and suggests a sex‐dependent effect. The results support the notion that in the treatment of dyslipidemia, both genetic and gender traits should be taken into account, and they point in the direction of personalized medical care, which is one of the goals of GWAS in clinical practice.

## AUTHOR CONTRIBUTIONS

**Dalal Al‐Sharshani** and **Alaa Rahhal** contributed to Writing—review and editing and formal analysis. **Dinesh Velayutham** contributed to formal analysis and software. **Muthanna Samara** contributed to writing—review and editing, formal analysis, software, and validation. **Mahmoud Ahmed** contributed to formal analysis. **Reham Gazal**, **Ayman Al Haj Zen**, **Mohamed A. Ismail**, **Gheyath Nasrallah**, **Salma Younes**, **Nasser Rizk**, **Sara Hammuda**, **M. Walid Qoronfleh**, **Thomas Farrell**, **Hatem Zayed**, **Palli Valapila Abdulrouf**, **Rana Al‐Jurf**, **Ahmed Mahfouz**, and **Amar Salam** contributed to writing—review and editing. **Hilal Al Rifai** contributed to writing—review and editing and resources. **Nader I. Al‐Dewik** contributed to conceptualization, data curation, formal analysis, investigation, methodology, resources, software, validation, visualization, writing—original draft, writing—review and editing, supervision, funding acquisition, and correspondence. All authors reviewed and approved the submission.

## FUNDING INFORMATION

This work was supported by the Qatar genome project and Qatar biobank.

## CONFLICT OF INTEREST STATEMENT

The authors declare that they have no known competing financial interests or personal relationships that could have appeared to influence the work reported in this paper.

## ETHICS STATEMENT

The study was approved by the Institutional Review Boards of the Qatar Biobank Protocol no. QF‐QBB‐RES‐ACC‐00018.

## Supporting information


Table S1.

Table S2.

## Data Availability

This is a research article and all data generated or analyzed during this study are included in this published article (and its supplementary information files). All enquiries should be directed to the corresponding author's Email: naldewik@hamad.qa; Nader.Al-Dewik@kingston.ac.uk.
