# Supplementary material for: Association of single nucleotide polymorphisms with dyslipidemia and risk of metabolic disorders in the State of Qatar
Source: Mol Genet Genomic Med. 2023 May 5;11(8):e2178. doi: 10.1002/mgg3.2178 (PMC10422074; doi:10.1002/mgg3.2178)
Supplement: Supplementary file 2 — Table S2. [file MGG3-11-e2178-s001.docx]

**Supplementary Table 2.** Logistic regression result for the six SNPs with covariates.

| **SNP** | **BP** | **A1** | **Test** | **NMISS** | **OR** | **STAT** | **P value** |  |
| --- | --- | --- | --- | --- | --- | --- | --- | --- |
| rs11172113 | 57133500 | C | ADD | 2101 | 0.8214 | -0.5682 | 0.5699 | |
|  |  |  | DOMDEV |  | 1.311 | 0.6034 | 0.5462 | |
|  |  |  | COV1 |  | 0.3426 | -6.948 | 3.71E-12 | |
|  |  |  | COV2 |  | 1.034 | 1.581 | 0.1139 | |
|  |  |  | COV3 |  | 1.05 | 1.707 | 0.08786 | |
|  |  |  | COV4 |  | 1.304 | 2.375 | 0.01756 | |
|  |  |  | ADDxCOV1 |  | 0.7498 | -1.457 | 0.1451 | |
|  |  |  | DOMDEVxCOV1 |  | 1.02 | 0.08003 | 0.9362 | |
|  |  |  | ADDxCOV2 |  | 1.01 | 0.4179 | 0.6761 | |
|  |  |  | DOMDEVxCOV2 |  | 1.014 | 0.4155 | 0.6778 | |
|  |  |  | ADDxCOV3 |  | 1.011 | 0.3092 | 0.7572 | |
|  |  |  | DOMDEVxCOV3 |  | 1.009 | 0.198 | 0.8431 | |
|  |  |  | ADDxCOV4 |  | 1.393 | 1.789 | 0.07363 | |
|  |  |  | DOMDEVxCOV4 |  | 0.8478 | -0.6977 | 0.4854 | |
|  |  |  | FULL_14DF |  | NA | 172.6 | 2.03E-29 | |
| rs646776 | 9275908 | C | ADD | 2101 | 1.387 | 0.6572 | 0.5111 | |
|  |  |  | DOMDEV |  | 0.3202 | -1.837 | 0.06619 | |
|  |  |  | COV1 |  | 0.2726 | -10.17 | 2.61E-24 | |
|  |  |  | COV2 |  | 1.029 | 1.605 | 0.1085 | |
|  |  |  | COV3 |  | 1.039 | 1.582 | 0.1137 | |
|  |  |  | COV4 |  | 1.49 | 3.705 | 0.000212 | |
|  |  |  | ADDxCOV1 |  | 0.7921 | -0.8011 | 0.4231 | |
|  |  |  | DOMDEVxCOV1 |  | 1.722 | 1.545 | 0.1223 | |
|  |  |  | ADDxCOV2 |  | 1.007 | 0.1745 | 0.8614 | |
|  |  |  | DOMDEVxCOV2 |  | 1.053 | 1.08 | 0.2802 | |
|  |  |  | ADDxCOV3 |  | 1.012 | 0.2635 | 0.7922 | |
|  |  |  | DOMDEVxCOV3 |  | 1.055 | 0.9224 | 0.3563 | |
|  |  |  | ADDxCOV4 |  | 0.9461 | -0.1993 | 0.842 | |
|  |  |  | DOMDEVxCOV4 |  | 0.9716 | -0.08854 | 0.9294 | |
|  |  |  | FULL_14DF |  | NA | 178.2 | 1.48E-30 | |
| rs1111875 | 92703125 | T | ADD | 2101 | 0.4775 | -2.201 | 0.02776 | |
|  |  |  | DOMDEV |  | 2.021 | 1.591 | 0.1116 | |
|  |  |  | COV1 |  | 0.2725 | -8.333 | 7.89E-17 | |
|  |  |  | COV2 |  | 1.067 | 3.081 | 0.002062 | |
|  |  |  | COV3 |  | 1.058 | 2.018 | 0.04362 | |
|  |  |  | COV4 |  | 1.299 | 1.921 | 0.05478 | |
|  |  |  | ADDxCOV1 |  | 1.234 | 1.2 | 0.2303 | |
|  |  |  | DOMDEVxCOV1 |  | 0.8451 | -0.7097 | 0.4779 | |
|  |  |  | ADDxCOV2 |  | 1.006 | 0.2652 | 0.7909 | |
|  |  |  | DOMDEVxCOV2 |  | 0.9513 | -1.545 | 0.1224 | |
|  |  |  | ADDxCOV3 |  | 0.9956 | -0.1328 | 0.8944 | |
|  |  |  | DOMDEVxCOV3 |  | 1.015 | 0.3383 | 0.7351 | |
|  |  |  | ADDxCOV4 |  | 1.235 | 1.291 | 0.1966 | |
|  |  |  | DOMDEVxCOV4 |  | 0.9082 | -0.4569 | 0.6477 | |
|  |  |  | FULL_14DF |  | NA | 176.3 | 3.60E-30 | |
| rs1801251 | 232768750 | A | ADD | 2101 | 1.386 | 1.223 | 0.2212 | |
|  |  |  | DOMDEV |  | 0.9975 | -0.00657 | 0.9948 | |
|  |  |  | COV1 |  | 0.3875 | -4.99 | 6.03E-07 | |
|  |  |  | COV2 |  | 1.052 | 1.902 | 0.05716 | |
|  |  |  | COV3 |  | 1.08 | 2.1 | 0.03573 | |
|  |  |  | COV4 |  | 1.395 | 2.218 | 0.02658 | |
|  |  |  | ADDxCOV1 |  | 0.6543 | -2.753 | 0.005899 | |
|  |  |  | DOMDEVxCOV1 |  | 1.206 | 0.8729 | 0.3827 | |
|  |  |  | ADDxCOV2 |  | 1.027 | 1.315 | 0.1885 | |
|  |  |  | DOMDEVxCOV2 |  | 0.9436 | -1.989 | 0.04667 | |
|  |  |  | ADDxCOV3 |  | 1.007 | 0.2322 | 0.8164 | |
|  |  |  | DOMDEVxCOV3 |  | 0.9498 | -1.305 | 0.1918 | |
|  |  |  | ADDxCOV4 |  | 1.018 | 0.145 | 0.8847 | |
|  |  |  | DOMDEVxCOV4 |  | 1.048 | 0.2584 | 0.7961 | |
|  |  |  | FULL_14DF |  | NA | 176.3 | 3.69E-30 | |
| rs2954029 | 125478730 | T | ADD | 2101 | 1.022 | 0.06828 | 0.9456 | |
|  |  |  | DOMDEV |  | 1.149 | 0.3118 | 0.7552 | |
|  |  |  | COV1 |  | 0.3189 | -7.886 | 3.11E-15 | |
|  |  |  | COV2 |  | 1.034 | 1.697 | 0.08974 | |
|  |  |  | COV3 |  | 1.054 | 2.009 | 0.04454 | |
|  |  |  | COV4 |  | 1.486 | 3.097 | 0.001952 | |
|  |  |  | ADDxCOV1 |  | 0.7642 | -1.371 | 0.1703 | |
|  |  |  | DOMDEVxCOV1 |  | 1.14 | 0.5035 | 0.6146 | |
|  |  |  | ADDxCOV2 |  | 0.9766 | -0.8474 | 0.3968 | |
|  |  |  | DOMDEVxCOV2 |  | 1.068 | 1.836 | 0.06632 | |
|  |  |  | ADDxCOV3 |  | 1.03 | 0.9421 | 0.3462 | |
|  |  |  | DOMDEVxCOV3 |  | 0.9721 | -0.6076 | 0.5435 | |
|  |  |  | ADDxCOV4 |  | 1.193 | 0.9899 | 0.3222 | |
|  |  |  | DOMDEVxCOV4 |  | 0.7083 | -1.509 | 0.1314 | |
| rs17514846 | 90873320 | A | ADD | 2101 | 0.5577 | -2.203 | 0.02756 | |
|  |  |  | DOMDEV |  | 3.2 | 2.998 | 0.002716 | |
|  |  |  | COV1 |  | 0.2779 | -6.261 | 3.82E-10 | |
|  |  |  | COV2 |  | 0.9887 | -0.3955 | 0.6925 | |
|  |  |  | COV3 |  | 1.023 | 0.6039 | 0.5459 | |
|  |  |  | COV4 |  | 1.668 | 2.5 | 0.01243 | |
|  |  |  | ADDxCOV1 |  | 1.298 | 1.803 | 0.07141 | |
|  |  |  | DOMDEVxCOV1 |  | 0.6103 | -2.326 | 0.02004 | |
|  |  |  | ADDxCOV2 |  | 1.07 | 3.429 | 0.000605 | |
|  |  |  | DOMDEVxCOV2 |  | 0.975 | -0.8682 | 0.3853 | |
|  |  |  | ADDxCOV3 |  | 1.011 | 0.4219 | 0.6731 | |
|  |  |  | DOMDEVxCOV3 |  | 1.055 | 1.355 | 0.1754 | |
|  |  |  | ADDxCOV4 |  | 0.9878 | -0.09191 | 0.9268 | |
|  |  |  | DOMDEVxCOV4 |  | 0.7322 | -1.646 | 0.09975 | |
|  |  |  | FULL_14DF |  | NA | 192.8 | 1.63E-33 | |

Abbreviations BP: Physical position (base-pair); A1: Tested allele (minor allele by default); ADD: additive effects of allele dosage; DOMDEV: dominance deviation from additivity, rather specifying that a particular allele is dominant or recessive; COV1: Gender; COV2: DM, COV3: HTN; COV4: Obese; GENO_2DF, a 2 df joint test of both additive and dominance; NMISS: Number of non-missing individuals included in analysis; OR odds ratio; STAT: Coefficient t-statistic; FULL_14DF: when adjusting for gender, obesity, hypertension and diabetes.
